# A Systematic Review of Research on Robot-Assisted Therapy for Children with Autism

**DOI:** 10.3390/s22030944

**Published:** 2022-01-26

**Authors:** Amal Alabdulkareem, Noura Alhakbani, Abeer Al-Nafjan

**Affiliations:** 1 Information Technology Department, College of Computer and Information Sciences, King Saud University, Riyadh 11543, Saudi Arabia; 438203344@student.ksu.edu.sa (A.A.); nhakbani@ksu.edu.sa (N.A.); 2 Computer Science Department, College of Computer and Information Sciences, Imam Muhammad bin Saud University, Riyadh 11432, Saudi Arabia

**Keywords:** robot-assisted therapy, autism spectrum disorder, assistive technology, systematic literature review

## Abstract

Recent studies have shown that children with autism may be interested in playing with an interactive robot. Moreover, the robot can engage these children in ways that demonstrate essential aspects of human interaction, guiding them in therapeutic sessions to practice more complex forms of interaction found in social human-to-human interactions. We review published articles on robot-assisted autism therapy (RAAT) to understand the trends in research on this type of therapy for children with autism and to provide practitioners and researchers with insights and possible future directions in the field. Specifically, we analyze 38 articles, all of which are refereed journal articles, that were indexed on Web of Science from 2009 onward, and discuss the distribution of the articles by publication year, article type, database and journal, research field, robot type, participant age range, and target behaviors. Overall, the results show considerable growth in the number of journal publications on RAAT, reflecting increased interest in the use of robot technology in autism therapy as a salient and legitimate research area. Factors, such as new advances in artificial intelligence techniques and machine learning, have spurred this growth.

## 1. Introduction

Autism spectrum disorder (ASD) is a neurological and developmental disorder that begins early in childhood. The Centers for Disease Control describe it as “a developmental disability that can cause significant social, communication and behavioral challenges”. Indeed, children with ASD face challenges that affect how they communicate, interact, behave, and learn. The specific development of their learning, cognition, and social skills varies by the child [1].

In clinical settings, diagnostic challenges can cause delays or misdiagnosis, such as limited knowledge of ASD among families as well as a lack of professionals and programs raising awareness of autism among the general public. Moreover, the ASD field lacks sufficient research, which can lead to insufficient quality of services being provided. Generally, delivering health care to children with ASD has many limitations given the small number of specialist centers and available professionals. Most importantly, diagnosing a person with ASD is difficult, and the process differs from one therapist to another. The medical center might also affect treatment and have high costs [2,3,4].

Several interventions have been developed for children with ASD with the aim of improving their cognitive ability and daily living skills, increasing their ability to function and participate in the community, and reducing symptoms. For instance, assistive technologies have been used during therapy sessions. This is driven by the societal need for new technologies that can facilitate and improve existing therapies for the growing number of children with ASD [5]. Such technologies can help children with ASD communicate and interact with others. One application is robot-assisted autism therapy (RAAT), which has been shown to be effective in various fields to support children’s developmental needs (e.g., sensory development, communication, interaction, cognitive development, social development, emotional development, and motor development) [6].

Socially assistive robotics is the research area on how robots assist people through social interaction [7]. It is a subfield of human-robot interaction (HRI), rehabilitation robotics, social robotics, and service robotics, with a focus on developing efficient interactions with the user in therapeutic and educational contexts [7]. Many researchers have developed robots to address the impairments and socially challenging areas faced by individuals with ASD, such as social learning, communication, interaction, imitation, and generalization skills [8].

Using robots during therapy sessions has benefits because individuals with ASD require the types of physical interaction that can be provided by robots since they have challenges communicating with real people [9,10]. Moreover, many studies have implemented RAAT solutions to help children with ASD develop social communication, education, and many other skills for their social world. For example, a robot was designed to teach music fundamentals to children with high-functioning autism [11]. In addition, researchers noted that long-term LEGO therapy with a humanoid robot for children with ASD significantly increased social initiations and had positive effects on the children’s engagement [12].

Yet, the use of social robots can present difficulties due to the heterogeneity of human populations. Lim et al. [13] examined cultural differences in how humans respond to robots designed to be socially assistive. Rudovic et al.’s [14] study on occupational therapy for autism suggested that Japanese children with ASD were more engaged with a socially assistive robot than were Serbian children with ASD.

Research in robot-assisted autism therapy cuts across diverse disciplines, including, but not limited to, developmental psychology, computer science, robotics, educational research, software engineering, and rehabilitation. These studies are published in various journals and various scientific domains, and they have different research focuses and methodologies. The main objective of this review is to classify and summarize research that is relevant to RAAT and to provide conceptual frameworks for integrating and classifying RAAT articles. This system of classification will be useful for literature reviews on RAAT research. This systematic review aims to address the following research questions: (i) Can robots effectively assist autism therapy? (ii) What are the challenges and future directions in the area of RAAT?

The following sections describe the method we applied for the review. Section 2 is an outline of the research methodology. Section 3 details the proposed classification framework for the literature review, and Section 4 presents a discussion. Section 5 offers insights for future research and discusses the challenges and trends in RAAT. Finally, Section 6 presents the study conclusions.

## 2. Research Methodology

Articles on robot-assisted therapy for children with ASD are scattered across conference proceedings and journals from various disciplines, including rehabilitation engineering, psychology, engineering, computer science, medical physics, and biomedical engineering. This section describes the procedure used for finding the relevant articles, along with the article-selection criteria and filtering processes.

**Figure 1:**
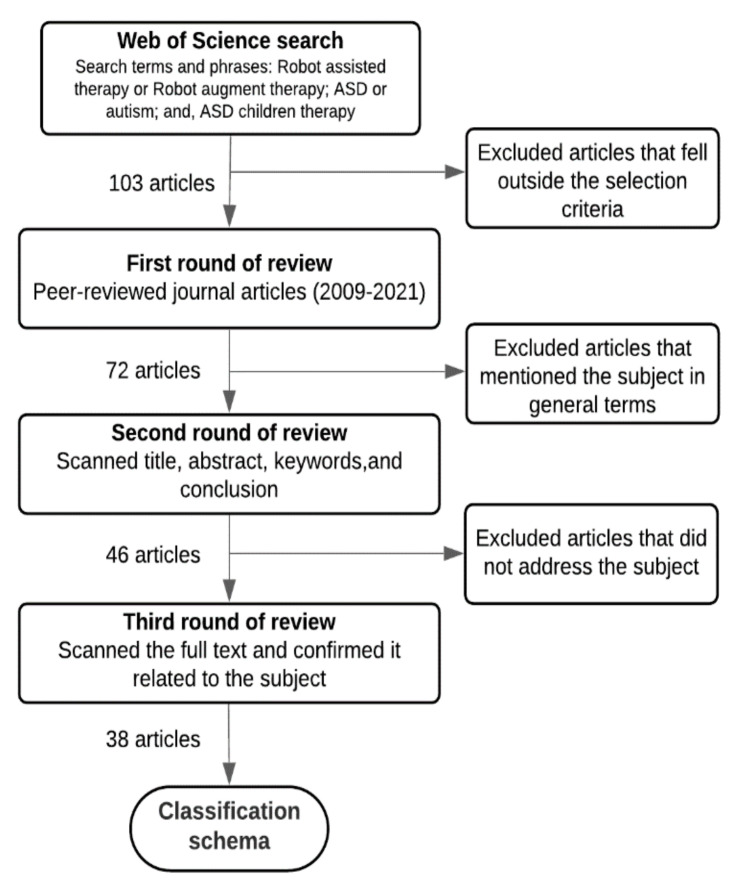
The procedure that was used to extract and filter articles.

We searched Web of Science (WoS) [15] to obtain a comprehensive bibliography of the academic literature on RAAT. The WoS database provides the most trusted, major world-leading citations from databases such as Science Direct (Elsevier), IEEE/IEE Library, ACM Digital Library, Springer Link Online Libraries, and Taylor & Francis. Specifically, we searched the WoS Core Collection database for articles over a span of 10 years, 2009–2021, Using basic search settings, we input search terms and phrases, such as: Robot-assisted therapy or Robot augmented therapy; ASD or autism; and, ASD children therapy. According to WoS search result templates, auto-generated search terms are a result of searches covering articles, meeting abstracts, book chapter(s), and proceedings papers. The initial search resulted in 103 research papers. From these, we selected only peer-reviewed journal articles and excluded meeting abstracts, book chapters, conference proceedings, workshop descriptions, masters and doctoral dissertations, and non-English language articles. Figure 1 shows the search results along with the filtering process.

In the first revision round, we omitted articles based on the previous selection criteria, which resulted in 73 research studies. These studies were imported into an online EndNote database to share, manage, and evaluate the articles. In the second revision round, we manually scanned the article titles, abstracts, keywords, and conclusions and removed articles whose fundamental research subject was not robot-assisted therapy or autism therapy. By the end of this round, we were left with 46 articles. The final round involved reading the full texts and analyzing each article. Finally, 38 articles remained for the analysis. After this filtering phase, we categorized each article based on our categorization scheme, detailed in Section 3.

## 3. Classification Method

We developed a literature-classification scheme to systematically reveal research insights on RAAT. The scheme was based on categorizing the research focuses of the 38 articles selected from among the 103 articles found in the initial search.

**Figure 2:**
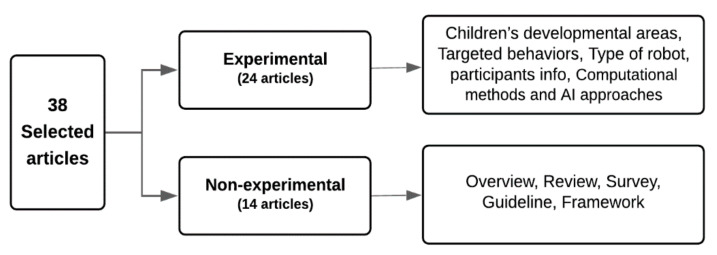
Classification of the selected articles.

In our review, we classified the articles by research orientation: experimental or non-experimental (see Figure 2). Next, we extracted additional information from each article, as explained in the following sections.

Of the articles, 63% (24 articles) were experimental. We classified them further into the following four groups:

- Children’s developmental areas: Involving processes that combined therapy with education to help children with ASD in their development (e.g., social and emotional, communication and interaction, cognitive, motor, and sensory development);
- Targeted behaviors: Therapeutic scenarios and activities that the robot could target (e.g., imitation, eye contact, turn-taking, emotion recognition and expression, self-initiated interactions, and/or triadic and dyadic interactions);
- Type of robot used in the concluded experiment (e.g., human-like, animal-like, toy-like, or machine-like robots) along with the name of the product (e.g., Nao robot, Kasper robot);
- Age and number of children who participated in the experiment.

Non-experimental articles propose a framework or guidelines; summarize the current state of a topic and related recent research papers; or conduct a survey, and then analyze the results and provide recommendations. Of the articles in this study, 37% (14 articles) were non-experimental. We classified them into five groups according to their focus:

- Overview articles: Summarize or recap either a product or ways to use it and include some other information, such as advantages and disadvantages or pros and cons;
- Review articles: Summarize the current state of some topics and the related recent research papers and create an understanding for the reader by discussing the findings, challenges, and future work;
- Survey articles: Collect information about a group of people (from interviews, questionnaires, focus groups, etc.) and then present and analyze the results and provide recommendations;
- Guideline articles: Provide guidelines for designing new solutions or alternative options to the current solution;
- Framework articles: Propose a structure intended to support or guide the design of something that expands the structure into something useful.

## 4. Results and Discussion

We extracted 38 articles that applied RAAT from 18 online databases covering 27 journals. Each article was reviewed and classified according to the classification scheme. 

Appendix A.1 and Appendix A.2 show trends in number of publications for robot-assisted autism therapy and distribution of our search results according to publication source and scientific activity field, respectively.

### 4.1. Experimental Papers

Of the articles, 63% (24 articles) were experimental. We classified these papers further according to their focus developmental areas.

#### 4.1.1. Article Classification by Children’s Developmental Area

Many processes combine therapy with education to help the development and growth of children with ASD. Ferrari et al. [16] identified therapeutic and educational objectives corresponding to children’s developmental areas: social and emotional development, communication and interaction development, cognitive development, motor development, sensory development, and areas other than developmental ones.

In our review, 56% of the articles (14 articles) fell into the social and emotional developmental area, 20% (five articles) were classified as communication and interaction development, 12% (three articles) related to cognitive development, and one paper was classified as motor-development skills. Also, one paper used a robot for screening children with ASD, which we determined to be outside our classification on developing skills for children with ASD. The following subsections explain what each area is and describe the articles in that category.

##### Social and Emotional Development

This is the domain in which the child experiences social situations, emotional expression, and engagement with others. The robot could improve the child’s sense of self, sharing and communication, and general comfort in joining others. As Table 1 shows, 56% (14 articles) were experimental papers based on social and emotional development.

**Social development** includes articles on building social skills for children with ASD. For example, Marino et al. [10] used a robot as an intervention mediator for social understanding. The results supported the use of the controlled assistive robot as an effective augmentative mediator in cognitive behavioral therapy protocols for social understanding.

**Emotional expression** includes articles on enhancing emotional expression for children with ASD. For example, Anamaria et al. [17] investigated whether the social robot Probo could enhance the children’s performance in identifying situation-based emotions. The results showed that the children’s performance in identifying both sadness and happiness improved with moderate to large effect sizes.

**Measuring engagement** includes articles on measuring engagement among children with ASD. For example, Rudovic et al. [14] measured engagement between two groups of children undergoing RAAT in a cross-cultural study. The results indicated statistically significant differences in engagement displays between the two groups; however, it is difficult to make any causal claims about these differences due to the large variations in the ages and behavioral severity of the children in the study. Moreover, Di Nuovo et al. [18] investigated novel deep-learning neural network architectures for automatically estimating if a child was focusing their visual attention on the robot during a therapy session.

**Table 1:** Social and emotional development articles.

Focus	Definition	Ref.
Social development	These articles investigate how selected robot features affect the development of social communication skills in children with ASD, with the aim of helping the children to understand and behave appropriately in social situations and improve their ability to generate and respond to behavioral requests	Marino et al., 2020 [10] Taheri et al., 2018 [19] Pop et al., 2013 [20] Mengoni et al., 2017 [21] Ghiglino et al., 2021 [22] Vanderborght et al., 2012 [23] Kim et al. 2021 [24] Yun et al., 2016 [25]
Measuring engagement	These articles measure engagement in RAAT by detecting their visual attention and positive changes in behaviors	Rakhymbayeva et al., 2021 [2] Rudovic et al., 2017 [14] Di Nuovo et al., 2018 [18] Rudovic et al., 2018 [26]
Emotional expression	These articles identify situation-based emotions with the aim of enhancing emotional expression among children with ASD	Anamaria et al., 2013 [17] Costescu et al., 2016 [27]

##### Communication and Interaction Development

Communication and interaction development involves providing children with basic communication skills. The robot could improve nonverbal aspects, such as eye contact and gestures (e.g., pointing, labeling, or touching), as well as verbal aspects, such as vocal communication. As Table 2 shows, 20% of the articles (five articles) were related to communication and interaction development.

**Verbal communication functionalities** include articles on building the verbal communication of children with ASD. For example, Lee et al. [28] investigated how selected robot features affected the development of social communication skills in children with low-functioning autism and studied their response to robots with verbal communication functionalities. The results showed that the children interacted with the verbal-featured robot more intensively than with the experimenter. Moreover, the authors concluded that the toy’s “face” and “moving limb” usually drew the children’s attention and improved their facial expression skills but did not contribute to the development of other social communication skills.

**Learning and interaction abilities** include articles on enhancing the learning and interaction abilities of children with ASD. For example, Bharataraj et al. [29] designed a parrot-inspired robot for this purpose. Their results indicated that the children were attracted to the robot and happy to interact with it.

**General interaction between robot and children** includes articles on the interaction abilities of children with ASD. For example, Boccanfuso et al. [30] employed a low-cost, toy-like robot prototype named CHARLIE. The authors designed and implemented interactive games to promote joint attention, imitation, and turn-taking. They found significant improvements in the children’s spontaneous utterances, social interaction, joint attention, and requesting behaviors. However, the overall communication scores, vocabulary, and motor imitation did not have statistically significant increases.

**Table 2:** Communication- and interaction-development articles.

Focus	Definition	Ref.
Verbal communication functionalities	These articles study the response of children with low-functioning autism to robots with verbal communication functionalities.	Lee et al., 2012 [28]
Learning and interaction abilities	These articles focus on behavioral development, including learning alphabets, numbers, human recognition, and assistance in other learning tasks.	Bharatharaj et al., 2017 [29] Bharatharaj et al., 2017 [31]
General interventions between robot and children	These articles focus on improving spontaneous utterances, social interaction, joint attention, and requesting behaviors.	Boccanfuso et al., 2017 [30] Silva et al., 2019 [32]

##### Cognitive Development

Cognitive development refers to mental processes that encompass the child’s ability to acquire knowledge and learn. A robot could improve memory functions, attention functions, imitation, and decision-making abilities. As Table 3 shows, 8% of the articles (three articles) were related to cognitive development.

**Proactivity and self-initiation** include articles enhancing proactivity and self-initiations. For example, van den Berk-Smeekens et al. [9] and Francois et al. [33] designed a robot-assisted therapy based on motivational components of pivotal response treatment focused on training pivotal key areas, such as motivation for social interaction and self-initiations, with the goal of establishing collateral gains in untargeted areas of functioning and development affected by ASD. The studies concluded that the children could adhere to the robot-assisted therapy protocol and showed positive affect ratings after therapy sessions.

**Perception enhancement** includes articles on perception. For example, Chen et al. [34] proposed a novel AI-based first-view-robot architecture. By providing care from the first-person perspective, the proposed wearable robot overcame the difficulties associated with the absence of cognitive ability in the third view of traditional robotics and improved the social interaction ability of children with ASD.

##### Motor Development

The motor-development area includes all aspects of body movements, as well as physical activities that involve both motor and psychological components (e.g., sequencing activities). For instance, a robot might improve body mobility movements and control of fine hand use. We found one paper presents an experiment on motor development. Palsbo and Hood-Szivek [35] explored the efficacy of robotic technology in improving handwriting in children with ASD who had impaired motor skills. The Probo robot showed positive effects in terms of increasing the children’s independence regarding social abilities.

##### Sensory Development

The sensory-development area relates to providing children with information and helping them understand their surrounding environment. In this area, a robot could improve visual perception, tactile perception, spatial awareness, and body awareness. We did not find any studies that fell into this category.

##### Areas Other than Developmental (Screening Children with ASD)

One article presented a study that used a parrot-like robot as a screening tool to diagnose children with ASD. There were 37 participants aged 2–11 years [36]. The paper described the design, development, and application of the robot as a screening tool to diagnose children with ASD. The results indicated it was effective, highlighting the importance of robot-assisted screening or intelligent toys in this field.

#### 4.1.2. Article Classification by Robot Type

For the experimental articles, we classified the type of robot used in the experiment into five categories: human-like, animal-like, toy-like, machine-like, and wearable robots; From our review, we found that the majority of the articles (ten articles) used a human-like robot in their studies [2,6,9,10,14,18,19,21,24,37], seven articles used an animal-like robot [27,28,29,31,32,33,36], five articles used a toy-like robot [17,20,22,23,30], two articles used a machine-like robot [25,35], and only one article used a wearable robot [34].

**Human-like robots** look like human beings, with a head, hands, and legs. In addition, the face contains eyes, a nose, and a mouth, and there are human external organs. We found that 40% of the articles (ten articles) used human-like robots. Nao, a well-known (in the market) human-like robot, was used in 32% of the studies (eight articles) [2,9,10,14,18,19,24,37]. Another human-like robot named Kasper was used in two studies [6,21].

**Animal-like robots** look like animals, commonly a dog, bird, or cat. The metal parts are usually covered with feathers or wool for appearance. We found that 28% of the articles (seven articles) used animal-like robots. KiliRo was used in 8% of the articles (two articles) [29,31], while Keepon [27], RoboParrot [36], ifbot [28], and Tornado [33] were used in one study each.

**Toy-like robots** come in many types with different shapes, sizes, and materials. They are mostly covered with feathers, wool, or fur. In addition, they sometimes contain facial and body parts to make them appear acceptable and friendly. We found that 20% of the articles (five articles) used toy-like robots. Probo was used in 12% of the articles (three articles) [17,20,23], while Cozmo [22] and CHARLIE [30] were used in one study each.

**Machine-like robots** look like electrical devices and come in different shapes and sizes. Usually, they are not covered with fabric. Two studies [25,35] used machine-like robots.

**Wearable robots** are worn on the human body. One study used a wearable robot [34].

#### 4.1.3. Article Classification by Participants

The number of children with ASD who participated in the experiments varied from 3 to 41 (mean = 18, median = 15, SD = 13, variance = 164), depending on the experiment type and field. The smallest number of participants was three [17]. In that study, the authors investigated whether the social robot Probo could enhance children’s performance in identifying situation-based emotions. The findings showed that children’s performance in identifying both sadness and happiness improved with moderate to large effect sizes.

The study with the largest number of participants had 41 children with ASD. Costecu et al. [27] investigated whether the children would demonstrate more irrational beliefs and therefore more dysfunctional emotions and maladaptive behaviors as compared to typically developing (TD) children. The authors proposed a robot-assisted task to test the hypothesis, and the results confirmed that children with ASD expressed significantly more irrational beliefs during the experimental tasks than their TD peers.

**Figure 3:**
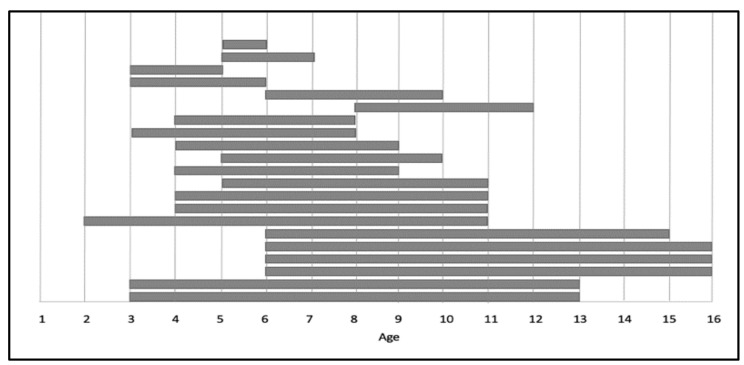
Demographic of participants’ age in experimental articles.

Moreover, as shown in Figure 3, different age groups participated in the experiments, ranging in total from 2 to 16 years old. Some of the studies involved wide age ranges. For example, three studies [24,29,31] had participants aged 6–16 years. Another two studies [14,26] were conducted with children aged 3–13 years. On the other hand, some of the experiments were conducted with narrow age groups. For example, Anamaria et al. [17] included participants aged 5–6 years old.

#### 4.1.4. Targeted Behaviors

Robots were used as mediators in the interaction sessions for several purposes, specifically to enable children with ASD to improve upon their impairments and communicate with the environment and people around them. The robots could target different therapeutic scenarios and activities with varying objectives and outcomes, such as imitation, eye contact, turn-taking, emotion recognition and expression, self-initiated interactions, and/or triadic and dyadic interactions. Table 4 shows the classification of papers based on target behaviors.

#### 4.1.5. Computational Methods and AI Approaches

This review reveals some weaknesses in the use and employment of AI in assistive robots for children with ASD. However, we noticed a good start in research measuring the engagement of children with ASD during therapy sessions using state-of-the-art computer vision, AI, and machine learning approach.

In the field of machine learning, two studies [18,37] applied a deep-learning approach to investigate the use of novel deep-learning neural network architectures for automatically estimating whether a child was focusing their visual attention on the robot during a therapy session, which is an indicator of their engagement. The researchers found that the proposed approach provided very high accuracy and is appropriate for use in assessment during therapy sessions.

Moreover, Rudovic and Picard [26] proposed a personalized machine learning framework for robot perception of affect and engagement in autism therapy. This framework supports the lack of ability to automatically perceive and respond to human affect, which is necessary for establishing and maintaining engaging interactions. The results of their experiment confirmed that robot perception of affect and engagement can be enhanced by personalizing the model. 

On the other hand, other studies applied subjective measurements like a coding approach for estimating engagement. For instance, in [14], researchers used a Likert scale to evaluate the level of engagement from evasive (non-compliance) to high engagement. Their coding was based on watching video recordings of robot-based interaction therapy, without employing any AI techniques. In [9], another subjective measurement was used for measuring the child’s affect and the likability of the robot. At the start and end of each robot-assisted session, the child was presented with a five-point Visual Analog Scale measuring child affect (i.e., “How happy are you now?”, with responses indicated by smiley faces).

### 4.2. Non-Experimental Papers

Among the articles, 37% (14 articles) were non-experimental. We classified them into the following five groups according to their focus: review, framework, overview, survey, and guidelines. Table 5 shows the distribution.

As a **review paper**, Yuan et al. [3] presented a systematic review of robotic rehabilitation for cognitive training. DiPietro et al. [5] reviewed the IT platforms being evaluated in computer- and robot-assisted social skills therapy for children with ASD, and they identified professional disciplines utilizing computer- and robot-assisted therapies, the outcomes, and the intervention activeness.

Four **framework papers** were found. Feng et al. [38] proposed a robotic architecture to improve the robot’s autonomy over the course of its interaction with children with ASD. Ueyama [39] proposed an emotional model of ASD that adapted a Bayesian model of the uncanny valley effect, which holds that a human-looking robot can provoke repulsion and sensations of eeriness. 

Baraka et al. [40] presented OAssistMe, an algorithm that generates cost-optimal action sequences given the action parameters. The authors instantiated their theoretical framework in the context of robot-assisted therapy tasks for children with ASD by determining action parameters based on a survey of domain experts and real child-robot interaction data. Cai et al. [41] attempted to improve the existing systems of both standard and robot-assisted therapy for children with ASD via a sensing framework with multi-sensory configuration and fusion.

Three **overview papers** provided general descriptions or summaries of the pros and cons of using robots in therapy. For example, Robins and Dautenhahn [42] provided an overview of how a robot can engage children with ASD in simple interactive activities or assume the role of social mediator. Wood et al. [43] discussed the development of Kaspar’s design and explained the rationale behind each change to the platform. Conti et al. [37] summarized the results of clinical and educational studies, showing the usefulness of social robots for supporting practitioners in their interventions with both TD children and children with neurodevelopmental disorders.

In a **survey paper**, Coeckelbergh et al. [44] presented a survey of expectations about the role of robots in robot-assisted therapy for children with ASD regarding ethical acceptability, trust, sociability, appearance, and attachment.

We found four **guideline papers**. Ramirez-Duque et al. [45] reported the development of a participatory design method aimed at identifying guidelines for designing a social robotic device to be implemented in robot-assisted therapy for children with ASD. Lytridis et al. [46] described synchronous and asynchronous therapeutic sessions as alternatives for children already participating in the protocol, in order to reduce the negative effects of the strict cessation of in-person sessions. 

Two studies presented guidelines for using robots in education. Huijnen et al. [4] explored how robots can be implemented practically into current education and therapy interventions for children with ASD. Robins and Dautenhahn [6] presented a set of tactile play scenarios, each with relevant educational and therapeutic objectives.

## 5. Challenges and Future Directions

**Figure 4:**
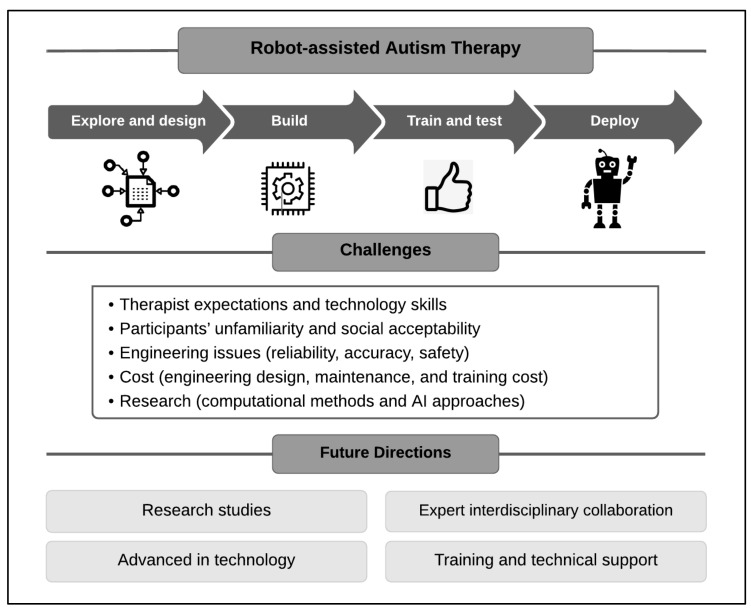
Challenges and future directions of robot-assisted autism therapy.

Research demonstrates that using robots can be effective in therapy sessions to motivate children with ASD to participate in activities, rather than traditional therapy sessions with their therapists [2,9,19,37]. Although the number of research studies on robot-based autism therapy has been increasing in recent years, it is still a new research area. Questions about the effectiveness and efficiency of using robots in autism therapy need to be addressed. Figure 4 provides insights into the challenges and future directions of robot-based autism therapy.

One challenge is that robots fall short of therapists’ expectations, which may result in them not being used effectively. To address this problem, a robot’s design and implementation require multidisciplinary skills from fields, such as engineering, computer science, psychology, and clinical rehabilitation. More experimental research studies are also needed to prove the benefits of robot use in autism therapy.

Moreover, there is a lack of therapists who are trained to work with robots that require assistance from the facilitator. This is one barrier that needs to be overcome by defining and standardizing training pathways. Another barrier to some interventions, besides cost, is the time and dedication that must be given to the robot-assisted intervention for the desired outcomes.

In addition, there are concerns about the social acceptability of robots and, more generally, about human-robot technologies as an effective tool for treating ASD. Engineers have questioned the design and choice of “robotic appearances”. Different design features and appearances have been suggested to increase the efficiency of therapy. In studying the potential therapeutic role of robots in autism therapy, as mentioned, researchers have used both humanoid robots (i.e., robots that have human-like appearances and perform at least the basic behaviors of human beings) and non-humanoid ones (i.e., robots that do not resemble humans).

The cost of robots is another barrier. However, although the initial costs (of engineering design, maintenance, and training) are high, a breakeven occurs relatively quickly. Some researchers have speculated that robot interventions have a high potential for making therapy more cost-effective.

Technological advancements in computational methods and AI approaches can be used to increase the effectiveness of robots. There is a need to create a robot-based autism framework to enable the development of this field using rigorous scientific methods. Some examples of limitations in current algorithms and approaches involve time constraints, accuracy, and reliability.

With recent technological advances, it becomes possible to develop advanced, reliable artificial robots for treating children with ASD. The robot should be able to interact with the child in a tailored manner and be adapted to their needs using AI techniques to guarantee successful autism therapy, because children with ASD behave differently from their TD peers.

Furthermore, AI and machine learning have been applied for automatically measuring the engagement of children with ASD. This allows the therapist to track a child’s engagement with ASD during therapy sessions without relying on traditional observation techniques. However, our research showed that this area has received attention in only a few studies. Accordingly, we recommend that researchers from different disciplines pay attention to future studies on this topic. In addition, we believe that more studies need to be conducted with robots in the field of clinical therapy, taking into account cost, accessibility, maintainability, training, adaptability, safety, and environmental concerns.

## 6. Conclusions

In recent years, many studies have explored the possible causes, rise in occurrence, and optimum interventions for ASD. Robot-assisted intervention should be viewed as a technological development that enhances the effectiveness and efficiency of autism therapy. Study findings demonstrate that improvements in treatments can play an important role in shaping new values of HRI related to human safety, efficacy, and cost-effectiveness.

This study reviewed published articles on RAAT. We identified 38 articles on the subject that were published in 2010–2019. Each article was reviewed, analyzed, and categorized according to its publication year, publication outlet, research type, and primary contribution.

This review helped us understand the various applied concepts of robots in autism therapy. Therefore, it could be considered as a reference source for researchers, academics, and practitioners interested in RAAT.

Based on the findings of this review, it is recommended to consider using robots in supervised applications to increase their acceptance and create more trust among therapists, children with ASD, and parents, as well as to improve the quality of therapy. However, more consideration should be given to addressing ethical and safety issues associated with these technologies.

We also found that the sensory development of children with autism needs more research to consider the use of robotics to improve the therapy. In addition, for targeted behaviors, we suggest that further research efforts need to focus on eye contact, cornering, self-initiated interactions, and triadic and dyadic interactions. Consequently, a collaboration between researchers, therapists, and medical practitioners is necessary to design and build an appropriate and beneficial robot to assist autism therapy. More research contributions are needed in artificial intelligence algorithms and computational methods to design and build this social intelligence robot for children with ASD. Moreover, a design guide needs to be set to improve the interaction between robots and ASD children.

Overall, we can assert that robot-assisted therapy is a promising field of application for intelligent social robots, especially to support children with ASD in achieving their therapeutic and educational objectives (social and emotional development, communication and interaction development, cognitive development, motor development, sensory development, and areas other than developmental ones). We anticipate that its challenges will be addressed in a timely manner with expert interdisciplinary collaboration.

## Figures and Tables

**Table 3:** Cognitive development articles.

Focus	Definition	Ref.
Proactivity and self-initiation	These articles focus on Pivotal Response Treatment (PRT), such as motivation for social interaction and self-initiations. Also, encouraging children’s creativity and initiative-taking.	Van den Berk-Smeekens et al., 2020 [9] Francois et al., 2009 [33]
Perception enhancement	These articles present cognitive wearable robotics for autism to improve perception.	Chen et al., 2021 [34]

**Table 4:** Target Behaviors.

Target Behaviors	Definition	Ref.
Imitation	The child’s ability to learn and develop new skills by copying others’ behaviors	Rakhymbayeva et al., 2021 [2] Taheri et al., 2018 [19] Mengoni et al., 2017 [21] Costescu et al., 2016 [27] Boccanfuso et al., 2017 [30]
Eye contact	A form of nonverbal communication and part of the child’s social development	Yun et al., 2016 [25] Conti et al., 2020 [37]
Joint attention	Attentional focus shared between two individuals looking at the same target using eye gaze or pointing	Taheri et al., 2018 [19] Mengoni et al., 2017 [21] Ghiglino et al., 2021 [22] Yun et al., 2016 [25] Boccanfuso et al., 2017 [30]
Turn-taking	The child’s ability to take turns and listen while another is speaking	Taheri et al., 2018 [19] Mengoni et al., 2017 [21]
Emotion recognition and expression	The child’s ability to read and interpret limited facial expressions and body language	Vanderborght et al., 2012 [23] Costescu et al., 2016 [27] Lee et al., 2012 [28] Bharatharaj, Huang, Elara, et al., 2017 [29]
Self-initiated interactions	The child’s ability to train themselves to initiate interactions and ask for things they need	Francois et al., 2009 [33]
Triadic and dyadic interactions	The triadic model uses a robot as a mediator between the child and other people, whereas the dyadic model only involves the robot and child	Marino et al., 2020 [10]

**Table 5:** Non-experimental articles classification.

Type	Definition	Ref.
Review	These articles review robotic rehabilitation for cognitive training and review the IT platforms evaluated and RAAT for ASD children.	Yuan et al., 2021 [3] DiPietro et al., 2019 [5]
Framework	These articles provide a model/framework or architecture in the context of robot-assisted therapy, for example, testing the effect of a human-looking robot using the Bayesian model. They also present the theoretical framework in the context of RAAT tasks for children with ASD and a propose sensing framework with multi-sensory configuration and fusion.	Feng, Jia, & Wei, 2018 [38] Ueyama, 2015 [39] Baraka, Melo, Couto, & Veloso, 2020 [40] Cai et al., 2019 [41]
Overview	These articles provide an overview of the ways in which the robot can engage autistic children. Also, they present an overview of projects on progress, such as Kaspar’s robot and CARER-AID projects. Both aimed at verifying the effects of the introduction of a humanoid robot in the clinical routine.	Conti et al., 2020 [37] Robins & Dautenhahn, 2018 [42] Wood, Zaraki, Robins, & Dautenhahn, 2021 [43]
Survey	These articles present a survey of expectations about the role of robots in robot-assisted therapy for children with ASD.	Coeckelbergh et al., 2016 [44]
Guidelines	These articles provide guidelines for the design of social robots to be implemented as RAAT for children with ASD. Moreover, some articles present an alternative to implementing synchronous and asynchronous therapeutic sessions.	Huijnen et al., 2017 [4] Robins & Dautenhahn, 2014 [6] Ramirez-Duque et al., 2021 [45] Lytridis et al., 2020 [46]

**Figure A1:**
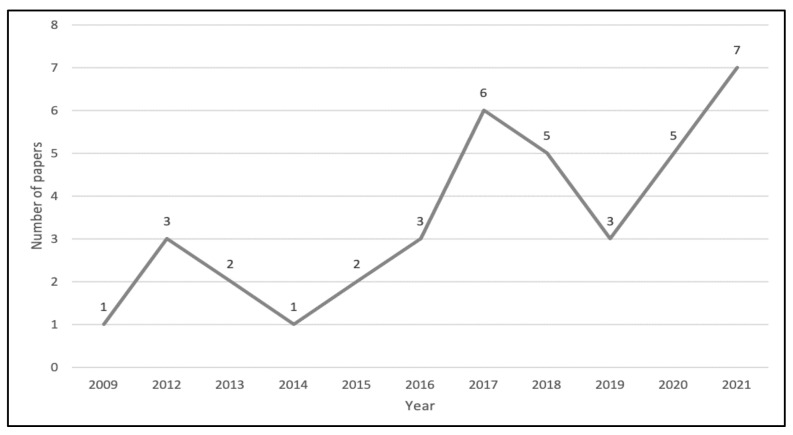
Number of papers published by year.

## Data Availability

Not applicable.

## Appendix A

### Appendix A.1. Trends in Number of Publications for Robot-Assisted Autism Therapy

Figure A1 is a line graph showing articles published from 2009 to 2021. Only 32% of the articles (12 articles) related to the subject were published in the period from 2009 to 2016. After that, the volume of publications rose sharply, culminating in a spike in 2017, comprising 16% of the total (six articles). By 2021, the number increased to 18% (seven articles).

### Appendix A.2. Publication Source and Scientific Activity Field

The article classifications by online database and journal are shown in Table A1 and Table A2, respectively. Table A1 lists the top 10 online article databases. Each of these hosted at least one article that matched the search criteria.

**Table A1:** Classification of articles by the online database.

Online Database	No. of Articles	Online Database	No. of Articles
Springer Nature	11	John Benjamins Publishing Co.	2
Frontiers Media Sa	4	Amer. Assoc. Advancement Science	1
MDPI	4	Amer. Occupational Therapy Assoc.	1
Sage	3	Assoc. Computing Machinery	1
Elsevier	2	BMJ Publishing Group	1

Table A2 shows the number of articles from each journal. We only documented journals with more than one published article. Most of the journals were related to robotics, developmental psychology, computer science (artificial intelligence; AI), multidisciplinary sciences, and automation control systems. The *International Journal of Social Robotics* had the largest number of articles, with 13% of the total (five articles), while *Frontiers in Robotics and AI* published 11% (four articles).

**Table A2:** Classification of articles by journal.

Journal	No. of Articles
International Journal of Social Robotics	5
Frontiers in Robotics and AI	4
Interaction Studies	2
International Journal of Advanced Robotic Systems	2
Journal of Autism and Developmental Disorders	2
Robotics	2

Table A3 shows the distribution of the WoS categorization results. Most of the articles were published in journals related to robotics, developmental psychology, and computer science (AI).

**Table A3:** Distribution of the Web of Science categorization results.

Field/Domain	No. of Articles	Field/Domain	No. of Articles
Robotics	18	Computer science (software engineering)	1
Developmental psychology	4	Educational research	1
Computer science (AI)	3	Special education	1
Multidisciplinary sciences	3	Multidisciplinary engineering	1
Automation control systems	2	Environmental studies	1
Communication	2	Ethics	1
Computer science (information systems)	2	History/philosophy of science	1
Electrical/electronic engineering	2	Instruments/instrumentation	1
Linguistics	2	Philosophy	1
General/internal medicine	2	Psychiatry	1
Applied physics	2	Clinical psychology	1
Rehabilitation	2	Respiratory system	1
Anthropology	1	Sociology	1
